# A Case Report of Guillain-Barré Syndrome Presenting as Facial Diplegia: A Deviation From the Classical Ascending Paralysis

**DOI:** 10.7759/cureus.101786

**Published:** 2026-01-18

**Authors:** Syed Abubacker, Yosif Albrahem, Hannah Shaw, Suhad Jalodi, Vikneswaran Raj Nagarajan, Asim Khaleeq, Syed Jafri

**Affiliations:** 1 General Internal Medicine, Kettering General Hospital NHS Foundation Trust, Kettering, GBR; 2 Acute Medicine, Kettering General Hospital NHS Foundation Trust, Kettering, GBR

**Keywords:** atypical guillain-barré syndrome, autoimmune neurology, bell’s palsy, bilateral facial nerve palsy, facial diplegia

## Abstract

Guillain-Barré syndrome (GBS) is an immune-mediated inflammatory disorder affecting the peripheral nerves, typically presenting with ascending paralysis and areflexia in the lower limbs, but can also have other variable presentations. Such variable presentations can rarely include an isolated involvement of the facial nerve as the initial presenting feature. We explore a case of a 53-year-old male patient who initially came with bilateral lower motor neuron facial nerve palsy, preliminarily diagnosed as inflammatory Bell’s palsy. However, the patient later re-presented with further neurological deficits involving the lower limbs, prompting further investigations that subsequently led to the diagnosis of GBS. This case highlights the importance of having a high index of suspicion for GBS when evaluating patients presenting solely with bilateral lower motor neuron facial nerve palsy, as GBS can rarely present in such a manner. While Bell’s palsy remains a common cause of facial palsy, bilateral involvement is unusual and should prompt evaluation for secondary causes such as GBS.

## Introduction

Guillain-Barré syndrome (GBS) is an immune-mediated inflammatory disorder affecting the peripheral nerves, characterised by the classical clinical picture of ascending paralysis affecting the lower and upper limbs, with potential progression to life-threatening respiratory muscle paralysis [1]. Bilateral facial nerve palsy is a rare presentation, accounting for around 0.3% to 2% of all facial nerve palsies [2]. While Bell’s palsy is a common cause of facial palsy, bilateral involvement is unusual and should prompt evaluation for secondary causes such as GBS, sarcoidosis, and Lyme disease [3]. A systematic review by Molinari et al. determined that the most frequent cause of bilateral facial nerve palsy is autoimmune [4]. Meanwhile, idiopathic Bell’s palsy is less frequently seen in people with facial diplegia as compared to unilateral presentations, prompting the need for extensive workup, which often results in a considerable diagnostic challenge [5].

The significance of documenting this case report lies in the rarity of GBS presenting initially as isolated facial diplegia. More frequent documentation of such rare presentations can lead to enhanced awareness among clinicians to recognise rare patterns of the disorder, prompt further investigations, and contribute to future literature as a more recognisable variant of GBS.

## Case presentation

This case follows a 53-year-old male van driver with no significant medical history apart from a previous history of trauma to his back and fused lumbar vertebrae. His symptoms initially started around March 2025, with generalised headache and loss of taste. He woke up the following day with bilateral facial numbness and the inability to make any facial expressions. He denied any recent respiratory or gastrointestinal symptoms suggestive of infections. There was no history of fever or earache.

On examination, cranial nerve examination was unremarkable apart from signs suggestive of bilateral lower motor neuron facial nerve palsy. The eye movements were normal. The right eye rolled upwards (more prominently than the left) on attempted closure. He was unable to make a smile or raise both eyebrows, and the sensation over the face was intact. Power was 5/5 in all limbs, and the tone was normal, as were the reflexes. Initial routine blood tests are shown in Table 1.

**Table 1:** Routine blood results during the first and second presentation. CRP: C-reactive protein; WBC: white blood cells; Hb: haemoglobin; eGFR: estimated glomerular filtration rate; MCV: mean corpuscular volume; PCV: packed cell volume.

Bloods	Results, 1^st^ presentation	Results, 2^nd^ presentation	Normal values
CRP	<5	<5	<5 mg/ L
WBC	11.5	15.6	4.0-11.0 x 10^9^/L
Hb	182	192	130-180 g/L
Platelets	391	452	150-450 x 10^9^/L
Urea	3.7	8.5	2.1-7.1 mmol/L
eGFR	>90	71.0	>90 ml/min/1.73m2
MCV	84.4	84.5	76-97 fL
PCV	0.552	0.540	0.400-0.540 L/L
Albumin	50	49	35-50 g/L
Total protein	77	77	60-80 g/L

**Figure 1:**
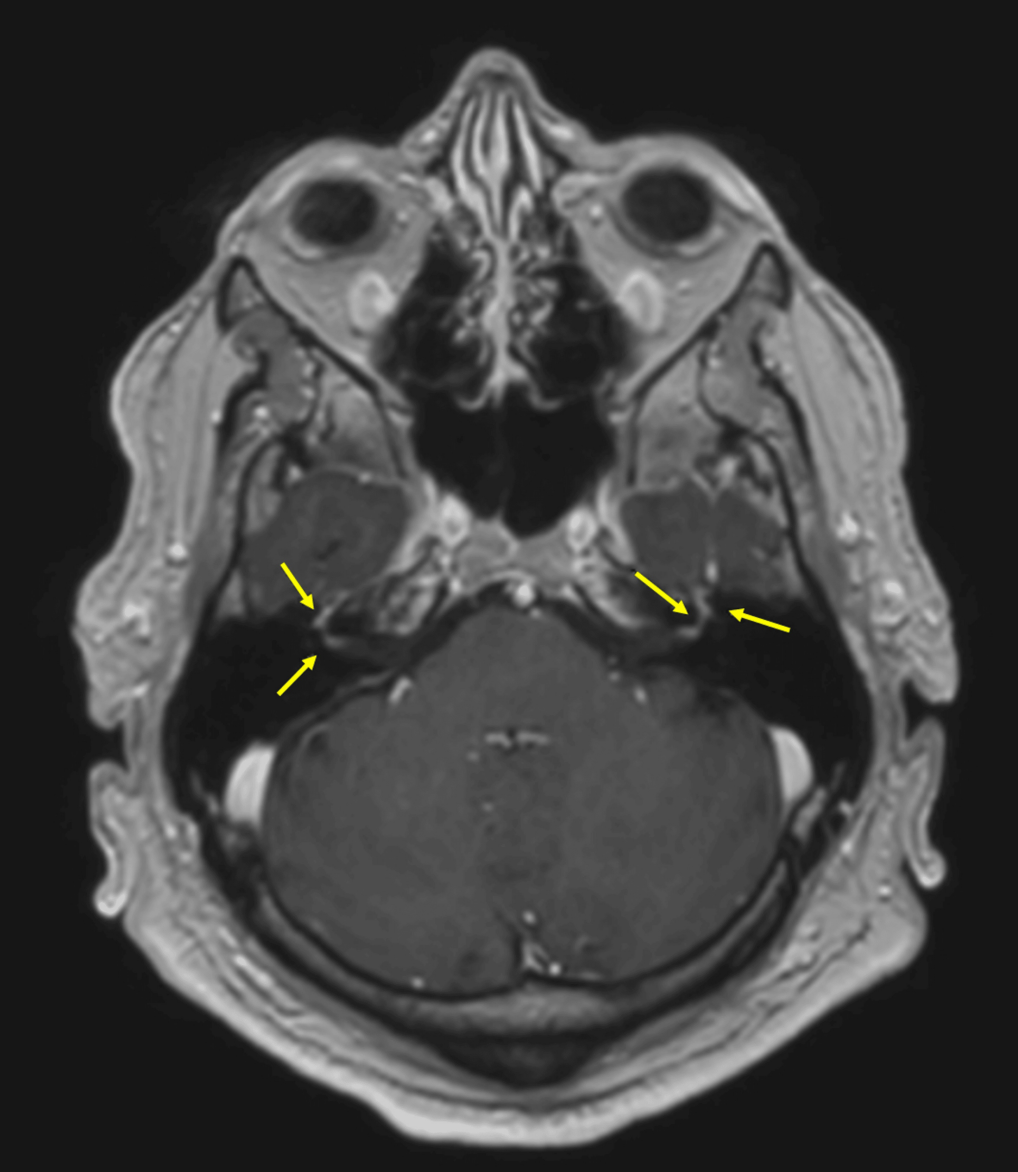
Axial section of MRI of the head with contrast showing pathologic enhancement at the fundus of both IAMs (yellow arrows). IAMs: internal acoustic meatuses.

He had a CT of the head and an MRI of the head that were both unremarkable. COVID-19 and influenza swabs were negative. He was treated for bilateral Bell’s palsy and started on a course of steroids. The patient had a neurology review due to the unusual nature of his presentation. The neurology team advised further investigations, including a lumbar puncture (LP), MRI of the head with contrast, with internal acoustic meatus (IAM) cuts, and further blood tests, including the glycolipid antibody markers, HIV, Venereal Disease Research Laboratory (VDRL) test, serum angiotensin converting enzyme (ACE) levels, acetylcholine antibodies, Lyme serology, anti-GQ1b antibody, and anti-MUSK (muscle-specific tyrosine kinase) antibody. He had multiple failed attempts at LP due to a previous history of trauma and fused vertebrae. The procedure was later planned under fluoroscopy guidance. However, MRI of the head with contrast showed pathologic enhancement at the fundus of both IAMs (Figure 1), which was consistent with inflammatory VII nerve palsy. The reason for the pathologic enhancement of the IAMs was reported as inflammation, which was initially deemed as Bell's palsy. Based on the contrast MRI findings, the patient was discharged home with steroids, valaciclovir, proton pump inhibitor (PPI) cover, and artificial tear drops, with the intention of outpatient neurology review.

However, he returned to the accident and emergency (A&E) five days later with ongoing bilateral facial nerve palsy and new onset of bilateral lower limb weakness associated with paraesthesia up to his mid-shins. Power in the lower limbs was recorded at 4/5, while the upper limbs remained normal. Reflexes in the lower limbs were noted to be diminished. An MRI of the whole spine was unremarkable. There was suspicion of GBS based on the progressive nature of the symptoms. This prompted a fluoroscopy-guided LP. His antibody screen panel, which was sent on the first admission, reported as negative, as shown in Table 2.

**Table 2:** Antibody screen panel results. ACHR: acetylcholine receptor; MUSK: muscle-specific tyrosine kinase.

Antibody screens and other immunological tests	Results
Glycolipid antibody screen	
GM1 IgG	Negative
GM1 IgM	Negative
GD1a IgG	Negative
GD1a IgM	Negative
GQ1b IgG	Negative
GQ1b IgM	Negative
GD1b IgG	Negative
GD1b IgM	Negative
GM2 IgG	Negative
GM2 IgM	Negative
Myasthenia antibodies	
ACHR antibodies	Negative
MUSK antibodies	Negative

Fluoroscopy-guided LP was performed, and results showed cyto-albuminic dissociation. The full results of the CSF analysis are shown in Table 3.

**Table 3:** Cerebrospinal fluid (CSF) analysis results.

CSF analysis	Results	Reference range
Cell count	WBC = 2 x 10^6^/L; RBC = 0 x 10^6^/L	-
Culture	No growth	
Protein/albumin	2.32	0.18-0.58 g/L
Glucose	3.9	2.8-4.4 mmol/L
Lactate	1.8	1.1-2.4 mmol/L

**Figure 2:**
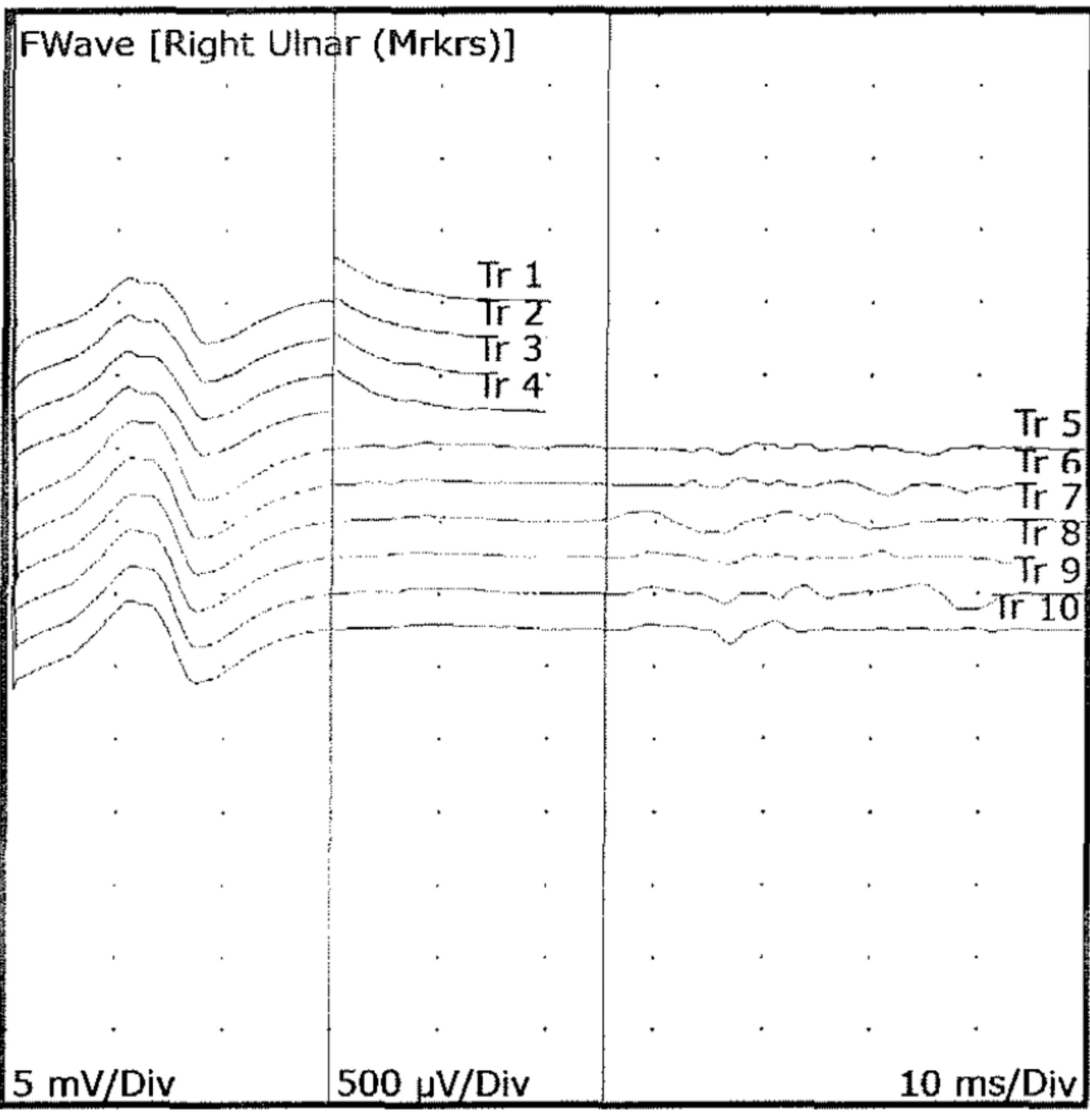
Nerve conduction study showing prolonged F waves.

Following another review from the neurology team, a diagnosis of GBS was made, and he was commenced on intravenous immunoglobulin (IVIG) on day 20 from the start of his initial symptoms and day 10 of the second hospital admission. HIV, *Treponema pallidum*, and *Borrelia burgdorferi* (Lyme disease) antibodies were all negative. Serum ACE was sent twice across both admissions, but the sample was haemolysed on both occasions, so the test was abandoned after confirmation of the diagnosis of GBS. The patient was monitored on the ward with daily forced vital capacity (FVC) measurements, which were satisfactory. Nerve conduction studies (NCS) then came back and showed prolonged F waves (Figure 2), supporting the diagnosis of GBS.

The patient was subsequently discharged home and followed up as an outpatient in the neurology clinic about three months later. At the neurology follow-up, he had shown significant improvement with regard to his symptoms of facial weakness, swallowing, and walking, although there was an incomplete resolution from his initial presentation. He still reported some difficulty closing his eyes and leg pain while walking. He was prescribed amitriptyline and was provided with an explanation and reassurance, with a plan to follow up in one year. Given that he had an established diagnosis of GBS with a demyelinating picture and that he had shown good improvement at the time he was seen in the clinic, the neurologist commented that they could anticipate ongoing or near-complete recovery.

## Discussion

While it is not uncommon for patients with GBS to have facial nerve involvement, with almost half of them affected [6], facial diplegia as the sole initial presenting feature of GBS is very rare, with less than 1% of cases reported to present in such a manner [7]. It can follow a preceding precipitant such as COVID-19 [8,9] or a flu-like illness [10]. In our case presentation, there was no history of antecedent infections or obvious triggering factors, suggesting that it can present without a prior trigger. Such a finding was also observed in a case report of GBS with facial diplegia without preceding infections described by Sardar et al. [11].

The pathophysiology of GBS involves immune-mediated damage affecting the peripheral nerves in response to certain infections or other triggers [12]. Molecular mimicry is one mechanism that can explain how GBS develops, where antibodies produced by B-cells cross-react with human antigens in the peripheral nerves that resemble antigens found on the lipo-oligosaccharide (LOS) molecules of certain bacteria, inducing an immune-mediated damage [13]. About two-thirds of patients with GBS report a recent gastrointestinal or respiratory infection or vaccination [13]. In the other one-third, there is no identifiable trigger, as described in our case report, which raises the question of whether there are other possible mechanisms involved that can explain the occurrence of GBS in these patients, something which has been described in the literature, detailing mechanisms involving T-cells and cytokines rather than B-cells and antibodies [14].

The implications of these immune mechanisms in the pathophysiology, and particularly autoantibodies, imply that IVIGs play a role in the management of GBS. Several studies found that IVIG and plasma exchange significantly hasten the recovery of symptoms in GBS, although they argued about which one is more effective than the other [15-17], while corticosteroids alone showed no significant impact on the disease [17]. In our case study, the patient received IVIG on day 20 of the onset of facial weakness, and subsequent neurology follow-up three months after symptoms onset showed good neurological improvement, which is consistent with the aforementioned literature, although the consensus among different guidelines recommended that IVIG needs to be administered within two weeks of the beginning of weakness [18-20].

Our case study described a rare presentation of GBS as bilateral lower motor neuron facial nerve palsy as the initial presenting feature. Other differential diagnoses were excluded in this case through history, examination findings, neuroimaging, serological tests, and subsequently CSF analysis and nerve conduction studies. It did show, however, the diagnostic difficulty of such a presentation and how GBS diagnosis can initially be missed in people presenting with facial diplegia, which has the impact of delaying appropriate treatment.

Limitations of our case study include the non-generalizability of our findings, given the nature of case reports. The pathophysiological and immune mechanisms described can only be inferred from existing literature due to the lack of histopathological studies and the negative status of antiganglioside antibodies in our case study. Nevertheless, it remains a clinically relevant case study that highlights the importance of having a high index of suspicion of GBS for any patient presenting with bilateral facial nerve palsy. Future research may focus on case series describing these rare presentations and literature reviews that adopt a diagnostic algorithm for facial diplegia that incorporates GBS as a main differential.

## Conclusions

While ascending paralysis is a hallmark of GBS, facial diplegia, despite being rare, is an important presenting feature of this disease that requires a high index of suspicion. It confers a significant diagnostic challenge and can be confused with Bell’s palsy. Therefore, early recognition and comprehensive neurological evaluation are crucial to avoid delayed treatment.
